# The Vehicle for Chloral

**Published:** 1872-05

**Authors:** 


### The Vehicle for Chloral.

The effect of chloral is modified by the vehicle in which it is administered. If given in syrup, it is absorbed slowly, and its effects are more prolonged. This is, consequently, the best form of vehicle when it is wished to procure a night's sleep. If given in a thinner vehicle, as camphor water or simple water, it shows its affects far more rapidly, and they pass off in a shorter time. So rapidly may chloral be decomposed in the blood, that the chloroform is let free with too great rapidity for safety.—*Virginia Clinical Record.*

